# The role of cytotoxicity in the process of carcinogenesis

**DOI:** 10.1007/s00204-026-04408-w

**Published:** 2026-04-28

**Authors:** Michael Schwarz, Bernd Epe, Laura E. Wohak, Carola Voss, Andrea Hartwig

**Affiliations:** 1 Department for Experimental and Clinical Pharmacology and Pharmacogenomics, Eberhard Karls University, Wilhelmstr. 56, 72074 Tübingen, Germany; 2 Institute of Pharmaceutical and Biomedical Sciences, Johannes Gutenberg University Mainz, Staudinger Weg 5, 55128 Mainz, Germany; 3 Institute of Applied Biosciences (IAB), Food Chemistry and Toxicology, Karlsruhe Institute of Technology (KIT), Adenauerring 20a, 76131 Karlsruhe, Germany; 4 Clinic for Cardiac, Thoracic, Transplantation and Vascular Surgery, Leibniz Research Laboratories for Biotechnology and Artificial Organs (LEBAO), Biomedical Research in Endstage and Obstructive Lung Disease Hannover (BREATH), German Center for Lung Research (DZL), Hannover Medical School, Carl Neuberg-Str. 1, 30625 Hannover, Germany

**Keywords:** Cytotoxicity, Carcinogenesis, Tumor promotion, ROS, DAMPs

## Abstract

Enhanced cancer prevalence findings restricted to high, cytotoxic dose levels in long-term animal cancer studies are generally assumed to be a consequence of—indirect or secondary—mutational effects, e.g. DNA damage mediated by generation of reactive oxygen species (ROS) or replication errors occurring during regenerative cell proliferation. An alternative explanation is provided by recent findings suggesting that cell lysis caused by cytotoxic doses of non-genotoxic agents may give rise to tumor promotion by selective growth stimulation of pre-existing (already mutated) dormant tumor precursor cells. This growth stimulation is assumed to be mediated by damage-associated molecular pattern (DAMP) signaling (“sterile inflammation”). Here, we discuss the differing views on cancer findings observed only at high cytotoxic doses in particular with respect to the implications for the risk assessment of the agents.

## Introduction

The German MAK Commission (German Senate Commission for the Investigation of Health Hazards of Chemical Compounds in the Work Area) is frequently confronted with the problem of cancer findings in experimental animals, which were exposed to very high doses of a chemical under investigation, at which cytotoxicity is also seen. In many of these cases, there are no indications for a mutagenic potential (direct genotoxicity) of the compound. Under these circumstances it is often assumed that cytotoxicity has impacted the process of carcinogenesis and thereby influenced the cancer outcome. In principle, two different mechanisms may play a major role in this process: (1) induction of indirect or secondary mutagenic effects (detailed below), (2) promotional (non-genotoxic) effects on already existent spontaneously mutated tumor precursor cells. For risk evaluation, it is important to understand which of these two quite different mechanisms is of major relevance in a human exposure setting. In this paper, we will discuss these aspects based on very recent findings reported in apparently unrelated studies, which become very relevant, however, when viewed within the context of the present question, namely (a), can cytotoxicity initiate the cancer process (by indirectly producing mutagenic effects), or (b) can cytotoxicity accelerate (promote) an already ongoing carcinogenic process or even act on later stages of tumor progression.

## Does cytotoxicity indirectly induce mutations via inflammation and oxidative stress?

The development of cancer involves one or more genetic alterations (mutations) in a target cell, which are passed on to all daughter cells and can, among other factors, be caused by DNA damage induced by chemically reactive agents (tumor initiation). In principle, cytotoxicity might cause mutations by two mechanisms: (1) Particularly at cytotoxic dose levels, damage to cellular targets other than DNA, e.g. the respiratory chain of mitochondria, can generate reactive cellular metabolites—such as reactive oxygen (ROS) and nitrogen species (NOS)—and thereby mutations, especially because cellular defense mechanisms (antioxidant response, DNA repair) may be malfunctioning at conditions of exhausted NADPH, ATP and GSH levels (“indirect genotoxicity”). (2) Alternatively, or in addition, tissue damage and cell death in vivo can either cause regenerative growth (associated with spontaneous mutations) or trigger an inflammatory response in which infiltrating neutrophiles and activated macrophages release ROS and potentially induce mutations in neighboring cells (“secondary genotoxicity”).

Evidence for indirect and secondary genotoxicity as a potential cause of tumorigenesis may be obtained by analyzing DNA damage spectra and mutational signatures in DNA, as ROS generate characteristic mutation patterns. The most characteristic DNA base modification caused by ROS and therefore oxidative stress is 8-oxo-2'-deoxyguanosine (8-oxoG). If not repaired by specific base excision repair initiated by 8-oxoguanine DNA glycosylase (OGG1), this leads to 8-oxoG:A mispairing. These inappropriately paired adenines can be excised by another specific DNA glycosylase (MUTYH). If both repair pathways fail this results in a specific type of base substitution, namely G > T/C > A transversions (Suzuki and Kamiya 2017, and references therein).

In cultured cells, the generation of 8-oxoG and its consequences have been studied after exposure to various types of oxidants. For example, potassium bromate quite selectively generates 8-oxoG resulting in a mutational spectrum dominated by G > T/C > A transversions (Kucab et al. 2019). Other examples are toxic/carcinogenic metal compounds. They may contribute to ROS-induced DNA damage and mutations by at least three distinct mechanisms: (1) redox-active metals ions like iron, copper or nickel catalyze Fenton-type reactions, (2) several metal compounds have been shown to inhibit DNA repair systems via specific interactions with distinct DNA repair enzymes, and (3) they may lead to GSH depletion at higher concentrations. Especially the second mechanism may contribute considerably to the extent of endogenous and carcinogen-induced DNA damage; one well documented example is arsenite, which specifically inhibits the activity of poly(ADP-ribose)polymerase at particularly low concentrations, thereby generating a repair deficient condition (Walter et al. 2007).

In animals, several xenobiotic oxidants have been shown to generate 8-oxoG, G > T/C > A mutations and ultimately cancer, e.g. potassium bromate (Arai, et al. 2002), ferric nitrilotriacetate (Jiang et al. 2006) and UVA irradiation (Ikehata et al. 2004). Information on the relevance of endogenous (spontaneously generated) DNA damage by ROS is obtained from *Ogg1* ^−/−^ mice which exhibit increased background levels of 8-oxoG and of spontaneous G > T/C > A mutations in the liver (transgenic *lacI* locus), but no significantly increased tumor incidence (Epe et al. 2002). However, after treatment with the peroxisome proliferator Wy-14,643, the number and volume of preneoplastic lesions in the liver of *Ogg1*^−/−^ mice were significantly increased compared to wild-type controls. This was not due to elevated ROS levels or additional ROS-induced DNA damage or mutations (Trapp et al. 2007). Instead, the enhancing effect on carcinogenesis in transgenic animals is likely due to the tumor-promoting properties of Wy-14,643, as discussed below. Interestingly, the additional knock-out of *Mutyh* gives rise to a strong increase of spontaneous tumors in lung, ovary and lymphatic cells of the mice, even without promoter treatment (Xie et al. 2004).

Although the findings described above confirm the potential of both exogenous and endogenous ROS generation to initiate tumors, several results suggest that the role of ROS in tumor formation is not primarily mediated by genotoxicity. Two chemically unrelated compounds, the phorbol ester TPA and hydrogen peroxide (H₂O₂) elicit a strong inflammatory response in mouse skin, which is accompanied by keratinocyte proliferation, epidermal thickening, and—ultimately -increased tumor yields (see e.g. Schwarz et al. 2013). This reaction is mediated, in part, by activation of immune-competent skin cells, which release both proliferation-promoting cytokines and ROS, particularly superoxide anions. The two agents, however, do not induce tumors in mouse skin when administered alone, without prior treatment with a tumor initiating agent. This contradicts the idea that ROS would trigger sufficient DNA damage for tumor initiation in this model. Rather, there is strong evidence that ROS act via non-genotoxic (i.e. promotional) mechanisms in the mouse skin model (see e.g., Schwarz et al. 1984). A compelling argument for the decisive role of ROS in this experimental setting comes from the observation that the synthetic superoxide dismutase mimetic CuDIPS completely inhibits tumor formation when chronically co-administered with TPA (Kensler et al. 1983).

If ROS-induced mutations were involved in papilloma formation, a corresponding ROS signature should be detectable in these tumors. In the two-stage initiation promotion experiment, where dimethylbenz(a)anthracene (DMBA) is administered as tumor initiator, the observed predominant signature, A > T/T > A transversions in codon 61 of the *Hras* oncogene, points to the reactive metabolite of DMBA as the causative agent. Additional mutational signatures that might be associated with ROS formation were also observed in this study. However, almost exclusively if DMBA exposure started in utero followed by delayed TPA treatment of the pubs (Li et al. 2025). Moreover, when TPA is administered only every two weeks, it loses its tumor-promoting effect (Boutwell 1964). This argues against a direct or ROS-mediated mutagenic mechanism, as such effects would be irreversible and cumulative, independent of the treatment schedule.

Regarding the relevance of oxidatively generated DNA damage (associated with inflammation) as mutagenic conditions at the origin of cancer in humans, important information is based on large-scale analyses, by which a range of characteristic mutational signatures have been identified across diverse human cancer types and in part assigned to causative agents or mechanisms (https://cancer.sanger.ac.uk/signatures/). Within this COSMIC database, Single Base Substitution (SBS) signature 18 is considered highly representative of a ROS-associated mutational profile, marked by a predominance of the expected G > T/C > A transversions. This ROS-specific signature was observed to varying degrees in multiple tumor types in an analysis of mutational patterns across more than 4500 fully sequenced human cancer genomes (Alexandrov et al. 2020). Moreover, cancers with a defect in the *MUTYH* gene display another signature (SBS36) which is qualitatively similar but quantitatively distinct to SBS18 (Pilati et al. 2017). The specific nucleotide context in which 8oxodG can lead to SBS18 and SBS36 has been elucidated using *OGG1* and *MUTYH* knockout cells (Zou et al. 2021).

Some human cancers are known to be strongly associated with chronic inflammation. These include a specific form of esophageal cancer that arises from intestinal metaplasia of the esophageal epithelium (Barrett’s esophagus, BE). In Western countries, this cancer type frequently develops as a consequence of chronic gastroesophageal reflux, which causes persistent irritation. A comparable situation is observed in ulcerative colitis (UC), a common chronic inflammatory bowel disease characterized by ongoing inflammation of the colon, recurrent ulcers, and in some cases, the development of colitis-associated colorectal carcinoma. Since ROS formation is a hallmark of inflammation, these two cancer types are very well suited to test the hypothesis that there might exist ROS-specific mutational signatures in these two cancers.

In crypts of UC patients the mutational landscape is however mainly characterized by T > G and T > C substitutions in the C[T]T/G[A]A context, which is associated with another mutational mechanism labeled SBS17(a/b) (Kakiuchi et al. 2020). A large-scale study on pre-cancerous BE, primary and metastatic esophageal tumors found that the majority of mutations during disease progression are attributable to SBS17a/b. SBS18 also contributed to mutation load, albeit to a lesser extent (Abbas et al. 2023). This confirmed data from a previous study on 97 sequenced esophageal cancers (Alexandrov et al. 2020). However, single-cell-sequencing analysis of low- to high-grade BE samples showed that SBS17-associated mutations only occurred in cells with chromosomal instability (CIN) (Busslinger et al 2021).

The mutational signatures enriched in both Barrett’s and UC tumor tissues, resembling SBS17a/b, suggest a link to chronic inflammation, which is therefore likely a causal factor in both diseases. However, the underlying mutagenic mechanism of Signature 17 remains unknown. Surprisingly, however, SBS18—associated with ROS—is underrepresented and not the major mutational signature enriched in these tissues. This raises doubts as to whether inflammation acts via ROS-mediated mutagenesis in a tumor-initiating (genotoxic) manner, i.e., through activation of oncogenes or inactivation of tumor suppressor genes.

In summary, ROS induced (oxidative stress related) mutations are clearly detectable in in vitro systems. In animals, a few oxidants have been shown to be mutagenic and to cause cancer. Also, the endogenous (spontaneous) generation of 8-oxoG contributes to tumor initiating events, and more so under repair-deficient conditions. This gives rise to carcinogenicity if combined with a tumor promoter or tumor promoting conditions. However, the mutational spectra observed in the two exemplary, clearly inflammation-associated human cancers do not provide solid evidence for an additional tumor-initiating (genotoxic) role of ROS in cancer development. In conclusion, since chronic inflammation clearly promotes tumor development, both in animals and humans, tumor-promoting non-genotoxic effects under chronically pro-inflammatory conditions may play a more significant role (see below).

## Does cytotoxicity induce mutations by stimulation of regenerative growth?

Mutations (and thus a potential initiating effect) following cytotoxic treatment may also arise through the induction of regenerative proliferation, as cell proliferation inherently carries a certain risk of spontaneous mutation. However, the available animal data argue against the hypothesis that increased proliferation alone leads to tumors. For example, a single or repeated partial hepatectomy (PH) in rats does not lead to neoplastic changes in the liver on its own. But when multiple PHs follow the administration of an initiating carcinogen, tumor development does occur, suggesting a promotional effect of PH (Pound and McGuire 1978). Similarly, a single necrotizing dose of carbon tetrachloride (CCl_4_) in mice induces regenerative hepatocyte proliferation without leading to fibrosis or subsequent liver neoplasms. Chronic application of necrotizing doses is required to induce fibrosis and tumors (Cohen et al. 2023). When CCl₄ is instead administered at four-week intervals in high single doses, the liver regenerates completely each time without subsequent fibrosis (see overview in Leist et al. 2017). This finding is reminiscent of the effects of TPA, whose tumor-promoting activity in mouse skin is completely lost when the same total dose, effective when applied twice weekly, is given intermittently with longer time intervals in between (Boutwell 1964).

Taken together, these findings suggest that neither the ROS-mediated indirect genotoxicity nor an induction of regenerative proliferation as such are as relevant for tumor formation, as often speculated. However, any mutation induced indirectly or secondarily via cytotoxicity will certainly add to the already existent mutational burden.

## How can tumor development be promoted by cytotoxic dose levels?

There is substantial evidence supporting a non-genotoxic, tumor-promoting role of cytotoxicity. A critical prerequisite for this mechanism is the pre-existence of tumor precursor cells within the regenerating tissue—cells that harbor spontaneously acquired mutations in oncogenes or other cancer-relevant genes.

One possibility is that a tumor-promoting activity of cytotoxicity results from its frequent association with inflammation and thereby ROS generation. ROS activate central transcription factors such as NF-κB, AP-1, and others. Moreover, many components of signal transduction pathways and transcription factors are redox-sensitive, meaning they can be modulated by ROS. Known redox-sensitive targets include MAP kinases, tyrosine phosphatases, APE1, TP53, and many more (Lennicke and Cochemé 2021; Averill-Bates 2024; Sies et al. 2024).

Another explanation for a tumor-promoting role of cytotoxicity, which is in the center of this paper, has recently been described for lung adenocarcinoma in never-smokers suspected to be linked to inhalation of airborne particulate matter in the nanometer range (PM2.5) (Hill et al. 2023). The proposed mechanism here involves a cascade of events initiated by cytotoxic effects within the lung target tissue.

To understand these effects, it is essential to briefly consider the innate immune system, whose primary function is the recognition of pathogens within the organism. Invasive pathogens display surface structures known as pathogen-associated molecular patterns (PAMPs), which are detected by specialized receptors on immune cells—so-called pattern recognition receptors (PRRs), with Toll-like receptors (TLRs) representing the largest subgroup. PRRs recognize conserved bacterial molecular structures and, upon binding, activate the immune system. Following lytic cell death—even in the absence of pathogens—intracellular components are released that act as damage-associated molecular patterns (DAMPs). One such DAMP is mitochondrial DNA (mtDNA), which, due to its evolutionary similarity to bacterial DNA, is recognized as “foreign” by certain PRRs. PRRs may also be activated by various proteins that are typically localized intracellularly but structurally resemble bacterial proteins, or by extracellular ATP (Gong et al. 2020).

These DAMPs (also known as alarmins) then activate, tissue-resident immune cells such as alveolar macrophages in the lung or Kupffer cells in the liver, releasing cytokines that recruit circulating immune cells. This amplifies the local immune response leading to an intensified inflammatory reaction. When no pathogens are involved, this response is referred to as sterile inflammation. For example, in the lung carbon-based nanomaterials from the environment can cause damage to respiratory epithelial cells, leading to the release of the DAMP interleukin (IL)-33. In addition, cell lysis (necroptosis) causes the depletion of alveolar macrophages and the release of the DAMP IL-1α. Together, these DAMPs reprogram the alveolar niche into a pro-inflammatory, pro-fibrotic environment, supported by the underlying activated mesenchyme. This environment is characterized by CCL2 (CC-chemokine-Ligand 2) expression and release, which attracts monocytes to the tissue, self-perpetuating persistent monocyte attraction supporting sustained inflammation. Accumulated inflammatory macrophages fail to replenish the alveolar space, sustaining the release of inflammatory cytokines (Voss et al. 2025). Among the mechanisms that trigger the release of DAMPs, ferroptosis, besides necroptosis, may play an important role (for review, see Huang et al. 2024). A comparable reaction, however, is probably less likely following apoptotic cell death, as this process typically does not result in the release of intracellular components. Instead, apoptotic cells form membrane-bound apoptotic bodies that emit a specific phospholipid signal to attract macrophages, instructing them to engulf and remove the dying cells (Lauber et al. 2003). In this way, an inflammatory reaction—such as that caused by lytic cell death—is effectively avoided.

While the processes described thus far represent protective host responses, the release of cytokines and cytokine-like signaling molecules can also have undesirable side effects. In particular, IL-1β released from attracted inflammatory macrophages has been shown to stimulate the proliferation of lung cells harboring dormant driver mutations in either epidermal growth factor receptor (*Egfr*) or *Kras* in a mouse model, thereby promoting tumor formation irrespective of the type of driver mutation (Hill et al. 2023). IL-1β from macrophages can also directly modulate the WNT pathway, promoting cancer cell proliferation (Kaler et al. 2009). A crucial assumption here is that a sufficient number of mutated tumor precursor cells preexist in the target tissue, having arisen through spontaneous or carcinogen-induced mutation. The presence of such cells is very likely, since cells from normal tissues from various human organs, including lung, have been shown to harbor hundreds to thousands of mutations, increasing linearly with age, and small clones of cells showing mutations in cancer driver genes (Fowler and Jones 2022; Yoshida et al. 2020; Lee-Six et al 2019; Brunner et al. 2019; Martincorena et al. 2015, 2018). Thus, dormant initiated cells may be subject to promotion (Li et al. 2025), in particular by an inflammatory response to cytotoxicity.

TLRs have been implicated in tumor promotion before. Fukata et al. (2007) reported, that Toll-like receptor-4 (TLR4) promotes the development of colitis associated colorectal tumors in humans. TLR7, on the other hand, has been demonstrated to play a decisive role in pancreatic carcinogenesis in mice and humans (Ochi et al. 2012). In diethylnitrosamine (DEN) exposed mice, IL-6 produced by Kupffer cells has been shown to stimulate hepatocarcinogenesis, an effect which was dependent on the TLR-adapter protein MyD88, ablation of which protected male mice from cancer development in their liver (Naugler et al. 2007). TLR4 deficient mice showed decreased IL-6 production in liver which resulted in attenuation of DEN induced hepatocarcinogenesis (Weber et al. 2016).

**Fig.1:**
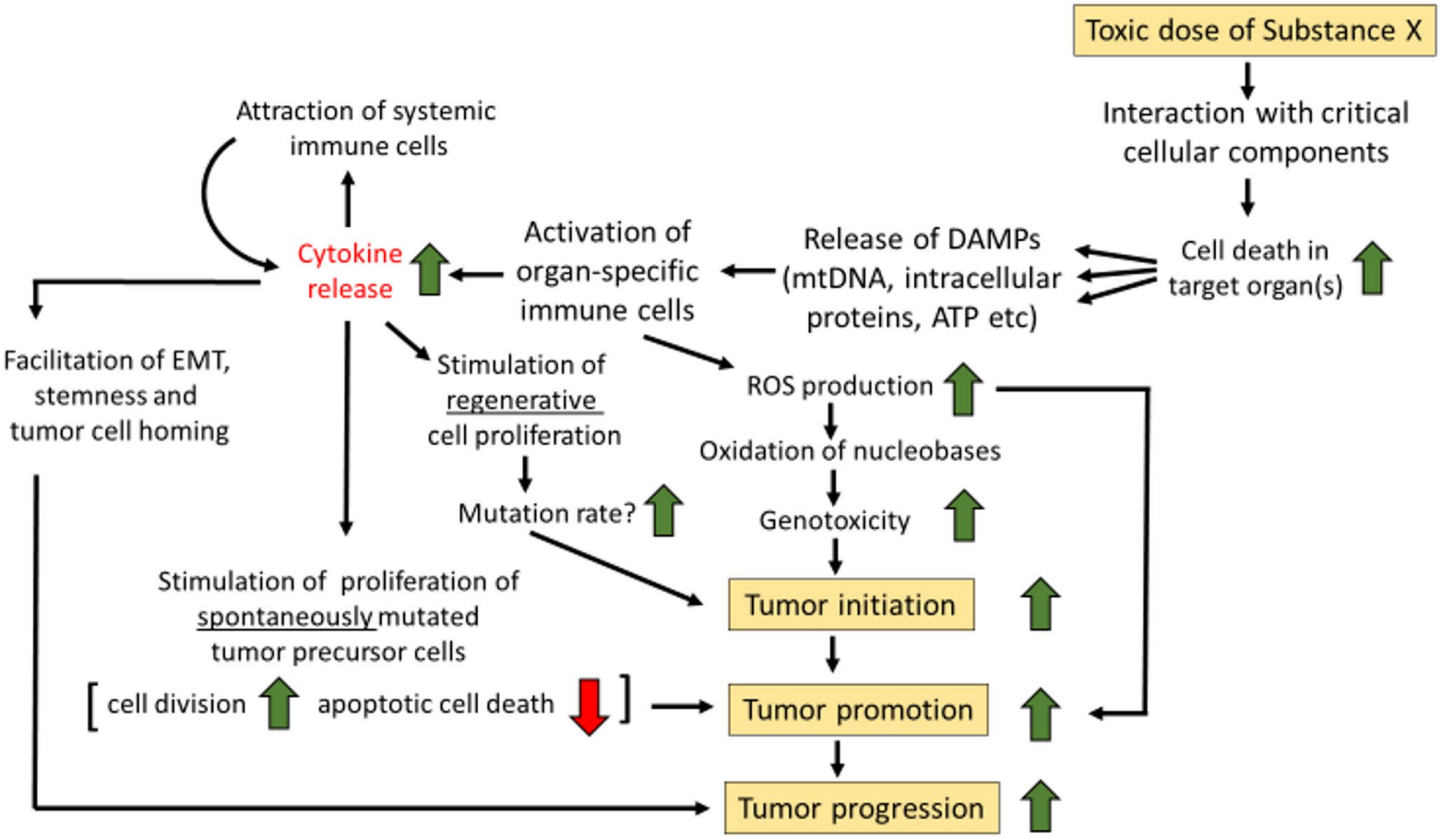
Schematic representation of the mechanisms by which xenobiotic-induced cytotoxicity in target tissues initiate or promote tumor development. *DAMPs* Damage-Associated Molecular Patterns; *mtDNA* mitochondrial DNA; *ROS* Reactive oxygen species; *EMT* Epithelial-to-Mesenchymal Transition

The chain of events described in this section is illustrated in Fig. 1, which also incorporates previously discussed (and deemed less relevant) tumor-initiating mechanisms at cytotoxic doses.

Other cytokine-like signaling molecules may also stimulate tumor cell proliferation. One such molecule is Wnt, which can be secreted by DAMP-activated immune cells (Bi et al. 2021). Wnt proteins are ligands for the so-called Frizzled receptors, and although they are not strictly classified as cytokines, they serve similar signaling functions within tissues. Wnt signaling molecules have been shown to stimulate tumor cell proliferation and inhibit their apoptosis both in vitro and in vivo (Xue et al. 2025).

Taken together, there is good evidence that cells carrying potential driver mutations remain dormant for very long periods of time, but become stimulated to proliferate when promoted by certain stimuli, in particular those associated with an inflammatory response to cell lysis. The mechanism appears to be related to that of several classical tumor promoters and in many cases explains the tumor incidences observed only at high (cytotoxic) doses of certain agents.

## Can cytotoxicity also affect the final stages of tumor development, including metastasis?

Figure 1 also illustrates another pathway through which cytotoxicity may indirectly influence tumor development, this time affecting the final stages of carcinogenesis, specifically tumor progression and metastasis. Once again, DAMP-stimulated immune cells play a central role through the release of cytokines or cytokine-like signaling molecules. These mediators can promote two key processes: first, the epithelial-to-mesenchymal transition (EMT), which is critical for metastasis; and second, the homing of metastatic tumor cells to distant sites, facilitating their survival and proliferation in a foreign microenvironment (Chaffer and Weinberg 2011).

The literature describes mechanisms by which these effects are mediated through Wnt molecules. In a study by Bi et al. (2021), mice exposed to graphene exhibited strongly enhanced tumor cell metastasis in the lungs, which was attributed to graphene-induced cytotoxicity in the target tissue, followed by a DAMP-driven immune response that included Wnt molecule release. ROS production was only minimally increased in alveolar macrophages in graphene exposed mice in this study and therefore excluded as source for the observed effects.

Other hypothetical examples may include the formation of intrahepatic metastases under chronic high-dose alcohol exposure or the promotion of distant metastases, e.g., in bone or lung tissue, by organ-specific toxins. However, these mechanisms remain largely speculative.

## Some important mechanistic considerations

At first glance, it may seem puzzling why a single, brief, or intermittent administration of a DAMP-inducing agent apparently fails to produce a detectable tumor-promoting effect. Theoretically, even such a transient stimulus should induce a temporary proliferation burst in spontaneously mutated tumor precursor cells, thereby increasing their number. Assuming this effect is irreversible, one would expect to observe cumulative effects with repeated applications, regardless of the duration of the interval between exposures. However, available animal data contradict this assumption.

Mechanistically, the explanation lies in the clonal nature of tumor development. Each cell within a clone has two possible fates: to proliferate or to undergo apoptotic cell death. Interestingly, tumor cells have a very high probability of dying, as first described by Kerr and Searle (1972). This holds also true for tumor precursor cells, especially while the clone is still small (see, e.g., Schulte-Hermann et al. 1993; Grasl-Graupp et al. 2000). As a result, most clones either die out or fail to expand significantly. This is precisely where tumor promoters intervene.

The best-studied example is hepatocarcinogenesis in rodents. In rats, for instance, 2,3,7,8-TCDD acts as a receptor-mediated tumor promoter in the liver. Its effect is primarily exerted not by stimulating cell division, but by inhibiting apoptosis of pre-neoplastic hepatocytes (Stinchcombe et al. 1995). Similar anti-apoptotic effects have been shown for numerous other tumor promoters.

A crucial point is that the anti-apoptotic activity is dependent on the continued presence of the tumor promoter. Once the promoter is withdrawn, the newly formed pre-neoplastic hepatocytes largely undergo apoptotic cell death (Schulte-Hermann et al. 1993). Therefore, chronic exposure is necessary to induce lasting effects.

## Implications for risk assessment of cytotoxic doses of non-genotoxic substances

The key conclusion from the preceding discussion is that cytotoxicity generally may not lead to tumor formation via indirect genotoxic mechanisms, but rather through non-genotoxic tumor-promoting activity, which appears to be the rate-limiting step in carcinogenesis. This has two important implications:

1. In cases of single or very short-term exposures that may well be cytotoxic, tumor-promoting effects are generally not expected. The induction of a *chronic* inflammatory response is a prerequisite for such effects to occur. The same applies to intermittent exposures where the individual exposure events are separated by long time intervals. It is likely that this also holds true for tumor progression effects, although solid evidence for this is currently lacking.
2. Dose–response (and time-response) relationships are non-linear, due to the underlying biological mechanisms.

Regarding the second point, three interconnected sub-processes must be considered individually (see also Fig. 1):

1. DAMP release in the target tissue
  This represents the initial event, which only occurs upon lytic cell death, i.e., at relatively high concentrations. At very low doses, cellular detoxification systems may be sufficient to buffer potential damage, thus preventing cell death altogether. With chronic exposure, damage accumulation is possible; however, adaptive protective mechanisms are also upregulated over time. In any case, the processes that ultimately lead to cell death are non-linear and are assumed to exhibit thresholds at low doses.
2. PRR activation by DAMPs
  Once DAMPs are released, pattern recognition receptors (PRRs) on local immune cells are activated, which then stimulate the immune response. This is a classic receptor-mediated response and can be expected to follow a non-linear dose–response relationship with a threshold.
3. Cytokine signaling in initiated tumor precursor cells
  The cytokines (or cytokine-like signaling molecules) released in response to PRR activation can engage surface receptors on “initiated” tumor precursor cells. These receptors relay pro-proliferative and anti-apoptotic signals into the cell. This, too, is a receptor-mediated process, and therefore subject to the same non-linear principles described for PRR activation.

In principle, step 3 in this chain of events resembles effects mediated by “classic” non-genotoxic tumor promoters such as phenobarbital, TCDD or TPA, which also mediate their effects as receptor ligands activating important cell-regulatory signal cascades involved in tumor promotion.

In summary, three sequential, non-linear processes underlie this mechanism. At high doses, they may amplify one another, while at low doses, they may dampen system responses—meaning that a few instances of lytic cell death alone are unlikely to trigger a consequential, full-blown inflammatory reaction or tumor promotion.

With respect to risk assessment it is important that *chronic* inflammation has to be induced to mediate tumor promotional effects and that these effects follow a non-linear dose–response relationship with a threshold.

## Abbreviations

DAMPs: Damage associated molecular patterns
IL: Interleukin
ROS: Reactive oxygen species
8-oxoG: 8-Oxo-7,8-dihydro-2'-deoxyguanosine
PRR: Pattern recognition receptor
TLR: Toll-like receptor
